# Supplementary material for: Heritable tumor cell division rate heterogeneity induces clonal dominance
Source: PLoS Comput Biol. 2018 Feb 12;14(2):e1005954. doi: 10.1371/journal.pcbi.1005954 (PMC5825147; doi:10.1371/journal.pcbi.1005954)
Supplement: S1 File — (PDF) [file pcbi.1005954.s006.pdf]

S1 File - Effect of spurious reads on clone loss and Gini coefficient

Quantification of cellular barcoding data depends on a complicated PCR and next-generation sequencing procedure, which is known to be error prone [1, 2]. Despite the development of powerful approaches to filter out such errors, it is expected that spurious reads still contaminate the final data. This implies that even when data are filtered based on a reference library of known barcodes, spurious reads that have been generated within these known barcodes are likely to occur. Such leakage would mean that some barcodes seem to be present that in reality are absent and the abundance of other barcodes is overestimated. However, spurious reads are not expected to be uniformly distributed over the clones because certain motifs are more likely to generate sequencing errors than others [3].

Therefore, we quantified the potential effect of spurious reads on the observed clone loss and Gini coefficient by adding spurious reads to our simulation results (Fig 1). By adding such noise to our simulation results we can test the effect that spurious reads are expected to have on our metrics. Because not all clones will be susceptible to gain spurious reads, we assign a percentage of clones as susceptible clones and randomly add the spurious reads to those clones. By varying the percentage of susceptible clones and the total amount of spurious reads that leaks into reference library clones we can study how sensitive clone loss and Gini coefficient are to such noise.

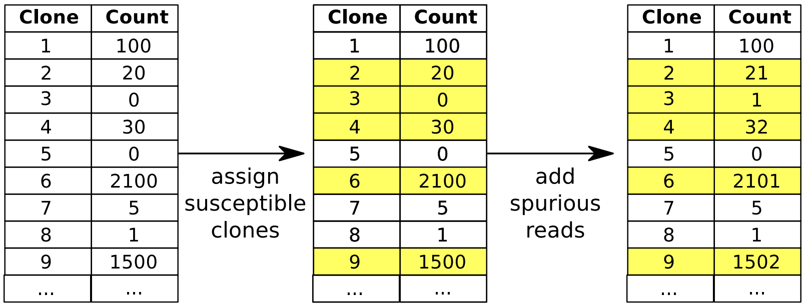

Fig 1. Schematic representation of the addition of spurious reads. Each table represents the clones (left columns) and their sizes (right columns). A subset of the clones is set to be susceptible for receiving spurious reads (highlighted in yellow) and the sizes of those clones are increased to simulate spurious reads.

To illustrate the potential effect of spurious reads, we applied this procedure to the results of a simulation initialized with the monoclonal K562 distribution, performed with parameters $\sigma_0^* = 0.006$ and $\sigma_m^* = 0.0006$ (Fig 2). Our results show that addition of spurious reads can increase the number of remaining clones to a level similar to that observed experimentally (Fig 2**A**). Note that the Gini

coefficient also increases due to spurious reads (Fig 2**B**) but this metric is less sensitive to spurious reads. Thus, it seems likely that the presence of spurious reads explains the difficulty our ABM has with matching the observed clone loss.

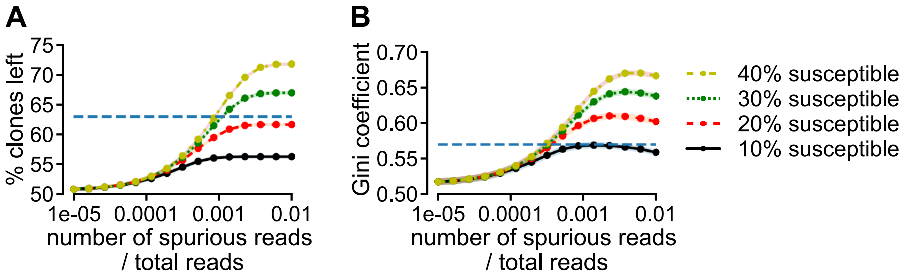

Fig 2. Spurious reads decrease clone loss and increase the Gini coefficient. A-B Percentage of remaining clones (**A**) and Gini coefficient (**B**) for an increasing number of added spurious reads. The spurious reads were added to the results of a simulation in which the initialization is based on the monoclonal K562 cells, with $\sigma_0^* = 0.006$ and $\sigma_m^* = 0.0006$. The horizontal dashed lines highlight the observed values for the monoclonal K562 cells. All data points are the mean of 10 simulations and shaded areas (only visible for the non-solid lines) represent the SD.

References

[1] Deakin CT, Deakin JJ, Ginn SL, Young P, Humphreys D, Suter CM, et al. Impact of next-generation sequencing error on analysis of barcoded plasmid libraries of known complexity and sequence. Nucleic Acids Res. 2014;42(16):e129. doi:10.1093/nar/gku607.

[2] Beltman JB, Urbanus J, Velds A, van Rooij N, Rohr JC, Naik SH, et al. Reproducibility of Illumina platform deep sequencing errors allows accurate determination of DNA barcodes in cells. BMC Bioinformatics. 2016;17:151. doi:10.1186/s12859-016-0999-4.

[3] Meacham F, Boffelli D, Dhahbi J, Martin DIK, Singer M, Pachter L. Identification and correction of systematic error in high-throughput sequence data. BMC Bioinformatics. 2011;12:451. doi:10.1186/1471-2105-12-451.
