# Supplementary material for: Heritable tumor cell division rate heterogeneity induces clonal dominance
Source: PLoS Comput Biol. 2018 Feb 12;14(2):e1005954. doi: 10.1371/journal.pcbi.1005954 (PMC5825147; doi:10.1371/journal.pcbi.1005954)
Supplement: S2 File — (PDF) [file pcbi.1005954.s007.pdf]

S2 File - Fitting the ABM describing division rate evolution to the HeLa data

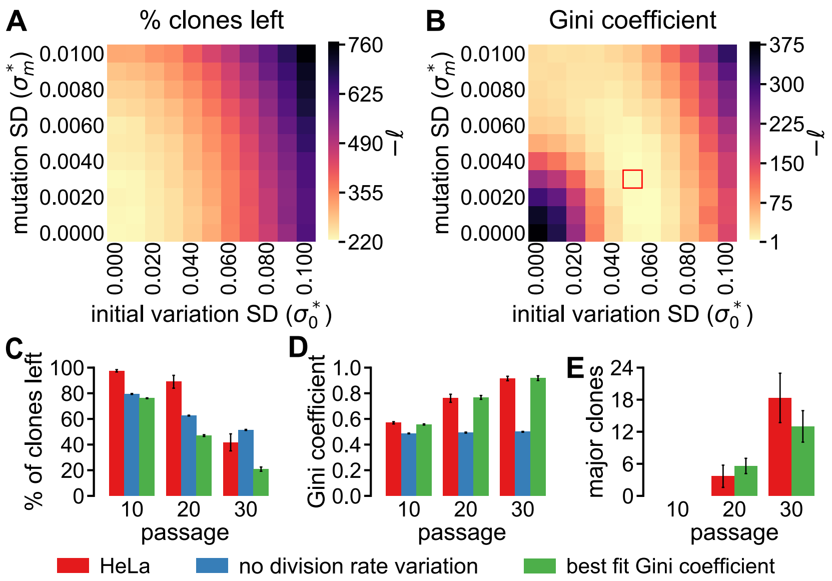

Fig 1. ABM simulations match clonal dominance development for the HeLa cell line. A-B Maximum Likelihood estimator (ℓ) for a range of initial division rate SDs (σ_0^*) and mutation SDs (σ_m^*), for either clone loss (**A**) or Gini coefficient (**B**). **C-E** Comparison of clone loss (**C**), clonal dominance (**D**) and number of major clones (**E**)for the *in vitro* experiments with HeLa cells, simulations without division rate variation, and simulations with the parameter set highlighted in **B**. All simulation results are the mean of 10 runs and the results for the HeLa cells are the mean of 3 replicates, with the error bars representing the SD.

We tested whether our model can also reproduce the clone size dynamics observed during *in vitro* growth and passage with HeLa cells. To this purpose, we ran a parameter sweep with each simulation initialized based on the initial clone size distribution for HeLa cells and performed an MLE with either clone loss (Fig 1**A**) or the Gini coefficient (Fig 1**B**). As with the monoclonal K562 cell line, no good match could be found when fitting to the clone loss data, but multiple parameter sets closely match the experimentally observed Gini coefficient data. Similarly, the best-fitting parameter set for the Gini coefficient results in an overestimation of clone loss (Fig 1**C**) while the Gini coefficient closely matches the *in vitro* observations (Fig 1**D**) as do the number of major clones (Fig 1**E**). The parameter set that produces the best fit for clone loss is $\sigma_0^* = \sigma_m^* = 0$, which corresponds to a model in which all cells divide at the same rate. However, this model still poorly matches clone loss and does not

induce any clonal dominance (Fig 1**C-D**). Given our results on the effect of spurious reads to the data, it seems likely that these explain at least a part of the mismatch with respect to clone loss.
