# Supplementary material for: Heritable tumor cell division rate heterogeneity induces clonal dominance
Source: PLoS Comput Biol. 2018 Feb 12;14(2):e1005954. doi: 10.1371/journal.pcbi.1005954 (PMC5825147; doi:10.1371/journal.pcbi.1005954)
Supplement: S3 File — File contains an executable jupyter notebook and a pdf print of that notebook as well as all code needed to process the FASTQ files. (ZIP) [file pcbi.1005954.s008.zip › S3_File/S1 File.html]

S1 File


In [17]:

```
%%capture
%pylab inline
%load_ext autoreload
%autoreload 2
import os,csv,gzip,copy
import pandas as pd
from IPython.display import display, HTML
from CloneTools import *

import matplotlib.gridspec as gridspec
import seaborn as sns

w_im = 3
h_im = 2.5
sns.set_style('ticks')
sns.set_context("notebook",font_scale=1,rc={'lines.linewidth' : 1.25})
sns.set_style('ticks',{'xtick.major.size' : 4,
                   'ytick.major.size' : 4})

datapath = '/home/mpalm/projects/tumhet_paper//expdata/'
```

# Obtaining metadata¶

To obtain the metadata for all files in SRP029299 at once we downloaded the mysql metadata database containing all metadata of the Sequence Read Archive ( http://dl.dropbox.com/u/51653511/SRAmetadb.sqlite.gz). After unzipping the database, we can read the database using Python's `sqlite3` module and extract the meta data we need. When we find this data, we store it in a text document for further usage.

In [4]:

```
import sqlite3

db = '/home/mpalm/Downloads/'+'SRAmetadb.sqlite'
study_acc = 'SRP029299'
info_tags = ['cell line', 'biological replicate','population doubling',
             'tag',"5' anchor","3' anchor"]
header = 'run accession\texperiment accession\t'+'\t'.join(info_tags)

# load database
conn = sqlite3.connect(db)

# get submission_accession for study
c = conn.execute("SELECT submission_accession FROM study WHERE study_accession=?",
                 (study_acc,))
sub_acc = c.fetchall()[0][0]

# get runs for study
c = conn.execute("SELECT run_accession,experiment_accession FROM run WHERE submission_accession=?",(sub_acc,))
runs = c.fetchall()
# get experiments for runs and write to file
df = pd.DataFrame(columns=['run accession','experiment accession','cell line', 
                           'biological replicate', 'population doubling', 
                           "5' anchor", "3' anchor", 'tag'])

for run_acc,exp_acc in runs:
    c = conn.execute("SELECT experiment_attribute FROM experiment WHERE experiment_accession=?",(exp_acc,))
    info = [item.split(':')[1].strip() for item in c.fetchall()[0][0].split(" || ")]    
    df = df.append(pd.DataFrame([[run_acc,exp_acc]+info],columns=df.columns))

df.to_csv('metadata_original.csv',sep='\t',index=False)
display(df)
```

|  | run accession | experiment accession | cell line | biological replicate | population doubling | 5' anchor | 3' anchor | tag |
| --- | --- | --- | --- | --- | --- | --- | --- | --- |
| 0 | SRR1211195 | SRX535199 | HeLa | A | 30 | GGCGCGCC | GCGG | GATC |
| 0 | SRR1211196 | SRX535200 | HeLa | A | 60 |  |  | CTAG |
| 0 | SRR1211197 | SRX535201 | HeLa | B | 30 | GGCGCGCC | GCGG | TGCA |
| 0 | SRR1211198 | SRX535202 | HeLa | B | 60 | GGCGCGCC | GCGG | GATC |
| 0 | SRR1211199 | SRX535203 | HeLa | C | 30 | GGCGCGCC | GCGG | ACGT |
| 0 | SRR1211200 | SRX535204 | HeLa | C | 60 | GGCGCGCC | GCGG | TGCA |
| 0 | SRR1211201 | SRX535205 | CCR5 targeting plasmid | n/a | n/a | CCTGCAGG | GGCG | ATCG, CGAT, GCTA, TAGC |
| 0 | SRR1211202 | SRX535206 | clonal K562 | A | 30, 60, 90 | GGCGCGCC | GCGG | CTAG, ACGT, TGCA |
| 0 | SRR1211203 | SRX535207 | clonal K562 | B | 30, 60, 90 | GGCGCGCC | GCGG | GATC, CTAG, ACGT |
| 0 | SRR1211204 | SRX535208 | clonal K562, clonal K562, clonal K562, CCR5 ta... | C, C, C, n/a | 30, 60, 90, n/a | GGCGCGCC, GGCGCGCC, GGCGCGCC, CCTGCAGG | GCGG, GCGG, GCGG, GGCG | TGCA, GATC, CTAG, ATCG |
| 0 | SRR1211205 | SRX535209 | HCC827 | n/a, A, B, C | 0, 30, 30, 30 | GGCGCGCC | GCGG | ACTG, CTAG, GATC, TGCA |
| 0 | SRR1211206 | SRX535210 | K562 | A, B, C | 30 | GGCGCGCC | GCGG | ACGT, CTAG, GATC |
| 0 | SRR1211207 | SRX535211 | K562 | A, B, C | 60 | GGCGCGCC | GCGG | ACGT, CTAG, GATC |
| 0 | SRR1211208 | SRX535212 | K562 | A, B, C | 90 | GGCGCGCC | GCGG | ACGT, CTAG, GATC |
| 0 | SRR1211209 | SRX535213 | plasmid library | n/a | n/a | GGCGCGCC | GCGG | ACGT, CTAG, GATC, TGCA |
| 0 | SRR1211210 | SRX535214 | K562, HeLa, HCC827 tumor 1, HCC827 tumor 2, ... | n/a | 0, 0, n/a, n/a, n/a | GGCGCGCC | GCGGCG, GCGG, GCGG, GCGG, GCGG, GCGG | CC, GATC, ACGT, CTAG, TGCA |
| 0 | SRR1211211 | SRX535215 | clonal K562, HCC827, HCC827, HCC827 | n/a, A, B, C | 0, 10, 10, 10 | GGCGCGCC | GCGG | CTAG, ACGT, GATC, TGCA |
| 0 | SRR1211212 | SRX535216 | 293T, HCC827, HCC827, HCC827 | n/a, A, B, C | 0, 20, 20, 20 | GGCGCGCC | GCGG | TGCA, ACGT, CTAG, GATC |
| 0 | SRR1211213 | SRX535217 | clonal K562, HeLa | n/a | 0 | GGCGCGCC | GCGG | TGCA, GATC |
| 0 | SRR1211214 | SRX535218 | CCR5 K562 | n/a | 0 | CCTGCAGG | GGCG | ATCG, CGAT, GCTA, TAGC |
| 0 | SRR1211215 | SRX535219 | CCR5 K562 | n/a, A, B, C | 0, 30, 30, 30 | CCTGCAGG | GGCG | TACG, AGTC, CTGA, GCAT |
| 0 | SRR1211216 | SRX535221 | CCR5 K562 | B | 60 | CCTGCAGG | GGCGCGCC | TACG |
| 0 | SRR1211217 | SRX535220 | CCR5 K562 | A | 60 | CCTGCAGG | GGCGCGCC | TACG |
| 0 | SRR1211218 | SRX535222 | CCR5 K562 | A, B, C, C | 90, 90, 60, 90 | CCTGCAGG | GGCG | AGTC, CTGA, TACG, GCAT |
| 0 | SRR1211219 | SRX535223 | CCR5 K562 | C | 60 | CCTGCAGG | GGCG | TACG |
| 0 | SRR1211220 | SRX535224 | CCR5 K562 | C | 60 | CCTGCAGG | GGCG | TACG |
| 0 | SRR1211221 | SRX535225 | CCR5 K562 | C | 60 | CCTGCAGG | GGCG | TACG |
| 0 | SRR1211222 | SRX535226 | 293T, 293T, 293T, HCC827 | A, B, C, n/a | 30, 30, 30, 0 | GGCGCGCC | GCGG | ACGT, CTAG, GATC, TGCA |
| 0 | SRR1211274 | SRX535227 | 293T | A, B, C | 30 | GGCGCGCC | GCGG | ACGT, CTAG, GATC |
| 0 | SRR1211275 | SRX535228 | 293T, 293T, HCC827 | A, B, A | 30, 30, 10 | GGCGCGCC | GCGG | ACGT, CTAG, TGCA |
| 0 | SRR1211276 | SRX535229 | 293T, 293T, 293T,293T, CCR5 K562, CCR5 K562 | A, A, B, B, A, C | 60, 90, 60, 90, 30, 30 | GGCGCGCC, GGCGCGCC, GGCGCGCC, GGCGCGCC, CCTGCA... | GCGG, GCGG, GCGG, GCGG, GGCG, GGCG | ACGT, CTAG, GATC, TGCA, AGTC, CTGA |
| 0 | SRR1211278 | SRX535230 | HeLa, HeLa, 293T, 293T, CCR5 K562, CCR5 K562 | A, B, C, C, A, C | 90, 90, 60, 90, 30, 30 | GGCGCGCC, GGCGCGCC, GGCGCGCC, GGCGCGCC, CCTGCA... | GCGG, GCGG, GCGG, GCGG, GGCG, GGCG | GATC, TGCA, ACGT, CTAG, AGTC, CTGA |
| 0 | SRR1211279 | SRX535231 | HeLa, CCR5 K562, CCR5 K562, HCC827, HCC827 | C, A, C, C, C | 90, 30, 30, 20, 30 | GGCGCGCC, CCTGCAGG, CCTGCAGG, GGCGCGCC, GGCGCGCC | GCGG, GGCG, GGCG, GCGG, GCGG | ACGT, AGTC, CTGA, GATC, TGCA |
| 0 | SRR1211280 | SRX535232 | HCC827 | C | 30 | GGCGCGCC | GCGG | TGCA |
| 0 | SRR1211282 | SRX535233 | HCC827 | B | 30 | GGCGCGCC | GCGG | ACGT |

The data shown above contains multiple entries per row. To make the data better accesible, we re-organize the data such that each row containts one entry.

In [5]:

```
df_new = pd.DataFrame(columns=df.columns)
for idx,row in df.iterrows():
    srr = row['run accession']
    srx = row['experiment accession']
    tags = row.tag.replace(' ','').split(',') 
    
    cell_lines = row['cell line'].replace(' ','').split(',')
    if len(cell_lines) == 1:
        cell_lines = len(tags)*cell_lines
    
    replicates = row['biological replicate'].replace(' ','').split(',')
    if len(replicates) == 1:
        replicates = len(tags)*replicates
    
    PD = row['population doubling'].replace(' ','').split(',')
    if len(PD) == 1:
        PD = len(tags)*PD
    
    begin_anchors = row["5' anchor"].replace(' ','').split(',')    
    if len(begin_anchors) == 1:
        begin_anchors = len(tags)*begin_anchors
    
    end_anchors = row["3' anchor"].replace(' ','').split(',')
    if len(end_anchors) == 1:
        end_anchors = len(tags)*end_anchors
    
    for i,t in enumerate(tags):
        df_new = df_new.append(pd.DataFrame([[srr,srx,cell_lines[i],replicates[i],
                                             PD[i],begin_anchors[i],end_anchors[i],t]],
                                            columns=df.columns))
                                             
df_new
```

Out[5]:

|  | run accession | experiment accession | cell line | biological replicate | population doubling | 5' anchor | 3' anchor | tag |
| --- | --- | --- | --- | --- | --- | --- | --- | --- |
| 0 | SRR1211195 | SRX535199 | HeLa | A | 30 | GGCGCGCC | GCGG | GATC |
| 0 | SRR1211196 | SRX535200 | HeLa | A | 60 |  |  | CTAG |
| 0 | SRR1211197 | SRX535201 | HeLa | B | 30 | GGCGCGCC | GCGG | TGCA |
| 0 | SRR1211198 | SRX535202 | HeLa | B | 60 | GGCGCGCC | GCGG | GATC |
| 0 | SRR1211199 | SRX535203 | HeLa | C | 30 | GGCGCGCC | GCGG | ACGT |
| 0 | SRR1211200 | SRX535204 | HeLa | C | 60 | GGCGCGCC | GCGG | TGCA |
| 0 | SRR1211201 | SRX535205 | CCR5targetingplasmid | n/a | n/a | CCTGCAGG | GGCG | ATCG |
| 0 | SRR1211201 | SRX535205 | CCR5targetingplasmid | n/a | n/a | CCTGCAGG | GGCG | CGAT |
| 0 | SRR1211201 | SRX535205 | CCR5targetingplasmid | n/a | n/a | CCTGCAGG | GGCG | GCTA |
| 0 | SRR1211201 | SRX535205 | CCR5targetingplasmid | n/a | n/a | CCTGCAGG | GGCG | TAGC |
| 0 | SRR1211202 | SRX535206 | clonalK562 | A | 30 | GGCGCGCC | GCGG | CTAG |
| 0 | SRR1211202 | SRX535206 | clonalK562 | A | 60 | GGCGCGCC | GCGG | ACGT |
| 0 | SRR1211202 | SRX535206 | clonalK562 | A | 90 | GGCGCGCC | GCGG | TGCA |
| 0 | SRR1211203 | SRX535207 | clonalK562 | B | 30 | GGCGCGCC | GCGG | GATC |
| 0 | SRR1211203 | SRX535207 | clonalK562 | B | 60 | GGCGCGCC | GCGG | CTAG |
| 0 | SRR1211203 | SRX535207 | clonalK562 | B | 90 | GGCGCGCC | GCGG | ACGT |
| 0 | SRR1211204 | SRX535208 | clonalK562 | C | 30 | GGCGCGCC | GCGG | TGCA |
| 0 | SRR1211204 | SRX535208 | clonalK562 | C | 60 | GGCGCGCC | GCGG | GATC |
| 0 | SRR1211204 | SRX535208 | clonalK562 | C | 90 | GGCGCGCC | GCGG | CTAG |
| 0 | SRR1211204 | SRX535208 | CCR5targetingplasmid | n/a | n/a | CCTGCAGG | GGCG | ATCG |
| 0 | SRR1211205 | SRX535209 | HCC827 | n/a | 0 | GGCGCGCC | GCGG | ACTG |
| 0 | SRR1211205 | SRX535209 | HCC827 | A | 30 | GGCGCGCC | GCGG | CTAG |
| 0 | SRR1211205 | SRX535209 | HCC827 | B | 30 | GGCGCGCC | GCGG | GATC |
| 0 | SRR1211205 | SRX535209 | HCC827 | C | 30 | GGCGCGCC | GCGG | TGCA |
| 0 | SRR1211206 | SRX535210 | K562 | A | 30 | GGCGCGCC | GCGG | ACGT |
| 0 | SRR1211206 | SRX535210 | K562 | B | 30 | GGCGCGCC | GCGG | CTAG |
| 0 | SRR1211206 | SRX535210 | K562 | C | 30 | GGCGCGCC | GCGG | GATC |
| 0 | SRR1211207 | SRX535211 | K562 | A | 60 | GGCGCGCC | GCGG | ACGT |
| 0 | SRR1211207 | SRX535211 | K562 | B | 60 | GGCGCGCC | GCGG | CTAG |
| 0 | SRR1211207 | SRX535211 | K562 | C | 60 | GGCGCGCC | GCGG | GATC |
| ... | ... | ... | ... | ... | ... | ... | ... | ... |
| 0 | SRR1211221 | SRX535225 | CCR5K562 | C | 60 | CCTGCAGG | GGCG | TACG |
| 0 | SRR1211222 | SRX535226 | 293T | A | 30 | GGCGCGCC | GCGG | ACGT |
| 0 | SRR1211222 | SRX535226 | 293T | B | 30 | GGCGCGCC | GCGG | CTAG |
| 0 | SRR1211222 | SRX535226 | 293T | C | 30 | GGCGCGCC | GCGG | GATC |
| 0 | SRR1211222 | SRX535226 | HCC827 | n/a | 0 | GGCGCGCC | GCGG | TGCA |
| 0 | SRR1211274 | SRX535227 | 293T | A | 30 | GGCGCGCC | GCGG | ACGT |
| 0 | SRR1211274 | SRX535227 | 293T | B | 30 | GGCGCGCC | GCGG | CTAG |
| 0 | SRR1211274 | SRX535227 | 293T | C | 30 | GGCGCGCC | GCGG | GATC |
| 0 | SRR1211275 | SRX535228 | 293T | A | 30 | GGCGCGCC | GCGG | ACGT |
| 0 | SRR1211275 | SRX535228 | 293T | B | 30 | GGCGCGCC | GCGG | CTAG |
| 0 | SRR1211275 | SRX535228 | HCC827 | A | 10 | GGCGCGCC | GCGG | TGCA |
| 0 | SRR1211276 | SRX535229 | 293T | A | 60 | GGCGCGCC | GCGG | ACGT |
| 0 | SRR1211276 | SRX535229 | 293T | A | 90 | GGCGCGCC | GCGG | CTAG |
| 0 | SRR1211276 | SRX535229 | 293T | B | 60 | GGCGCGCC | GCGG | GATC |
| 0 | SRR1211276 | SRX535229 | 293T | B | 90 | GGCGCGCC | GCGG | TGCA |
| 0 | SRR1211276 | SRX535229 | CCR5K562 | A | 30 | CCTGCAGG | GGCG | AGTC |
| 0 | SRR1211276 | SRX535229 | CCR5K562 | C | 30 | CCTGCAGG | GGCG | CTGA |
| 0 | SRR1211278 | SRX535230 | HeLa | A | 90 | GGCGCGCC | GCGG | GATC |
| 0 | SRR1211278 | SRX535230 | HeLa | B | 90 | GGCGCGCC | GCGG | TGCA |
| 0 | SRR1211278 | SRX535230 | 293T | C | 60 | GGCGCGCC | GCGG | ACGT |
| 0 | SRR1211278 | SRX535230 | 293T | C | 90 | GGCGCGCC | GCGG | CTAG |
| 0 | SRR1211278 | SRX535230 | CCR5K562 | A | 30 | CCTGCAGG | GGCG | AGTC |
| 0 | SRR1211278 | SRX535230 | CCR5K562 | C | 30 | CCTGCAGG | GGCG | CTGA |
| 0 | SRR1211279 | SRX535231 | HeLa | C | 90 | GGCGCGCC | GCGG | ACGT |
| 0 | SRR1211279 | SRX535231 | CCR5K562 | A | 30 | CCTGCAGG | GGCG | AGTC |
| 0 | SRR1211279 | SRX535231 | CCR5K562 | C | 30 | CCTGCAGG | GGCG | CTGA |
| 0 | SRR1211279 | SRX535231 | HCC827 | C | 20 | GGCGCGCC | GCGG | GATC |
| 0 | SRR1211279 | SRX535231 | HCC827 | C | 30 | GGCGCGCC | GCGG | TGCA |
| 0 | SRR1211280 | SRX535232 | HCC827 | C | 30 | GGCGCGCC | GCGG | TGCA |
| 0 | SRR1211282 | SRX535233 | HCC827 | B | 30 | GGCGCGCC | GCGG | ACGT |

98 rows × 8 columns

One row - for population doubling 60, replicate A of the HeLa cell line - is missing the 3' and 5' anchors. We assume that these anchors are the same as those for the other HeLa fields, and fix the data accordingly. Furthermore, we found that if we process run SRR1211210 with GCGGCG as the 3' anchor, we get too few results. Changing the anchor to the default (GCGGCC) fixed this, so we change that as well:

In [6]:

```
df_new.loc[df_new["run accession"]=='SRR1211196', "5' anchor"] = 'GGCGCGCC'
df_new.loc[df_new["run accession"]=='SRR1211196', "3' anchor"] = 'GCGG'
df_new.loc[df_new["3' anchor"]=='GCGGCG', "3' anchor"] = 'GCGGCC'
#df_new.loc[df_new["run accession"]=='SRR1211210', "3' anchor"] = 'GCGGCC'
```

Finally, we save the resulting data to file.

In [7]:

```
df_new.to_csv('metadata.csv',sep='\t',index=False)
```

# Processing FASTQ files¶

We processed the FASTQ files using our own C++ code, using bioio to read the FASTQ files. Briefly, the code opens each FASTQ files and then, for each set of tag and anchor in the file, finds the barcodes for which all bases meet the minimum qualtiy. The resulting barcodes are then counted and these barcodes and counts are writter to file, one file for each tag in each FASTQ file. Using the metadata file generated above, all FASTQ file can be processed with a single command: `process_all_fastq -m metadata.csv` (with a minimum quality of 63).

In the following we assume that:

1. ClusterSeq has been run with the default settings and all results are stored in `datapath/clusterseq/` (where `datapath` should be specified at the top of this notebook)
2. `process_all_fastq -m metadata.csv` has been run and the results are stored in `datapath/noclustering/q=63`
3. `process_all_fastq -q 56 -m metadata.csv` has been run and the results are stored in `datapath/noclustering/q=56`

In [8]:

```
def compare_methods(cell_line):
    df_reads = pd.DataFrame(columns=['run','tag','cell line','replicate','PD','reads porter','reads palm'])
    for idx,row in metadata[metadata['cell line']==cell_line].iterrows():
        run = row['run accession']
        tag = row['tag']
        data = [run,tag,row['cell line'],row['biological replicate'],
                row['population doubling'],count_reads_porter(run,tag),count_reads_palm(run,tag)]
        df_reads = df_reads.append(pd.DataFrame([data],columns=df_reads.columns))
    display(df_reads)
    
def count_reads_palm(run,tag,qual=63):
    fn = '{}/noclustering/q={}/{}_{}.txt.gz'.format(datapath,qual,run,tag)
    reader = csv.reader(gzip.open(fn),delimiter='\t')
    csvdata = {row[0]:int(row[1]) for row in reader if (not row[0].startswith('#'))}
    return np.sum(csvdata.values())

def count_reads_porter(run,tag):
    fn = '{}/clusterseq/{}.{}_clusters.csv.gz'.format(datapath,run,tag)
    reader = csv.reader(gzip.open(fn),delimiter=',')
    csvdata = {row[0]:int(row[1]) for row in reader if (not row[0].startswith('#'))}
    return np.sum(csvdata.values())
```

To ensure that we do not filter out too much data we compare the number of reads, i.e. the sum of all clones size, obtained with our code to that obtained with ClusterSeq. As shown below, we need to reduce the minimum quality to obtain a similar number of reads.

In [9]:

```
cell_lines = ['K562','HeLa','clonalK562','293T']
df_reads = pd.DataFrame(columns=['run','tag','cell line','replicate','PD','reads porter',
                                     'reads palm - q=63','reads palm - q=56'])


metadata = pd.read_csv('metadata.csv',sep='\t',index_col=False)

for idx,row in metadata[metadata['cell line'].isin(cell_lines)].iterrows():
    run = row['run accession']
    tag = row['tag']
    data = [run,tag,row['cell line'],row['biological replicate'],
            row['population doubling'],count_reads_porter(run,tag),
            count_reads_palm(run,tag,qual=63),count_reads_palm(run,tag,qual=56)]
    df_reads = df_reads.append(pd.DataFrame([data],columns=df_reads.columns))
display(df_reads)
```

|  | run | tag | cell line | replicate | PD | reads porter | reads palm - q=63 | reads palm - q=56 |
| --- | --- | --- | --- | --- | --- | --- | --- | --- |
| 0 | SRR1211195 | GATC | HeLa | A | 30 | 3487267 | 3064092 | 3281690 |
| 0 | SRR1211196 | CTAG | HeLa | A | 60 | 5106806 | 4698592 | 5033028 |
| 0 | SRR1211197 | TGCA | HeLa | B | 30 | 4602426 | 4146549 | 4526447 |
| 0 | SRR1211198 | GATC | HeLa | B | 60 | 3882515 | 3480109 | 3815929 |
| 0 | SRR1211199 | ACGT | HeLa | C | 30 | 4658322 | 4218358 | 4590101 |
| 0 | SRR1211200 | TGCA | HeLa | C | 60 | 5178330 | 4661663 | 5072077 |
| 0 | SRR1211202 | CTAG | clonalK562 | A | 30 | 4035342 | 1181401 | 3898698 |
| 0 | SRR1211202 | ACGT | clonalK562 | A | 60 | 4668850 | 1772509 | 4518307 |
| 0 | SRR1211202 | TGCA | clonalK562 | A | 90 | 3822106 | 1351965 | 3691537 |
| 0 | SRR1211203 | GATC | clonalK562 | B | 30 | 5516610 | 4087468 | 5406409 |
| 0 | SRR1211203 | CTAG | clonalK562 | B | 60 | 5983486 | 4500340 | 5874696 |
| 0 | SRR1211203 | ACGT | clonalK562 | B | 90 | 4782201 | 3606118 | 4683748 |
| 0 | SRR1211204 | TGCA | clonalK562 | C | 30 | 5577116 | 4764794 | 5464726 |
| 0 | SRR1211204 | GATC | clonalK562 | C | 60 | 5333009 | 4470629 | 5221458 |
| 0 | SRR1211204 | CTAG | clonalK562 | C | 90 | 4353121 | 3837394 | 4279171 |
| 0 | SRR1211206 | ACGT | K562 | A | 30 | 4169570 | 3713446 | 4087626 |
| 0 | SRR1211206 | CTAG | K562 | B | 30 | 3986086 | 3525433 | 3910252 |
| 0 | SRR1211206 | GATC | K562 | C | 30 | 6383216 | 5630814 | 6260574 |
| 0 | SRR1211207 | ACGT | K562 | A | 60 | 4564748 | 4107791 | 4494607 |
| 0 | SRR1211207 | CTAG | K562 | B | 60 | 4409766 | 3957719 | 4348984 |
| 0 | SRR1211207 | GATC | K562 | C | 60 | 4410671 | 3934819 | 4340003 |
| 0 | SRR1211208 | ACGT | K562 | A | 90 | 3439334 | 3064003 | 3387882 |
| 0 | SRR1211208 | CTAG | K562 | B | 90 | 3869988 | 3374697 | 3814973 |
| 0 | SRR1211208 | GATC | K562 | C | 90 | 4185563 | 3640101 | 4122418 |
| 0 | SRR1211210 | CC | K562 | n/a | 0 | 5921568 | 5128949 | 5742008 |
| 0 | SRR1211210 | GATC | HeLa | n/a | 0 | 1214946 | 1083229 | 1186301 |
| 0 | SRR1211211 | CTAG | clonalK562 | n/a | 0 | 2978711 | 114396 | 2290356 |
| 0 | SRR1211212 | TGCA | 293T | n/a | 0 | 7120896 | 5050780 | 6811984 |
| 0 | SRR1211213 | TGCA | clonalK562 | n/a | 0 | 3457390 | 2733155 | 3203585 |
| 0 | SRR1211213 | GATC | HeLa | n/a | 0 | 3898041 | 2976408 | 3599229 |
| 0 | SRR1211222 | ACGT | 293T | A | 30 | 1761153 | 814291 | 1437254 |
| 0 | SRR1211222 | CTAG | 293T | B | 30 | 2406717 | 1350396 | 2103292 |
| 0 | SRR1211222 | GATC | 293T | C | 30 | 2316164 | 1285484 | 2010756 |
| 0 | SRR1211274 | ACGT | 293T | A | 30 | 2172207 | 1393599 | 1959247 |
| 0 | SRR1211274 | CTAG | 293T | B | 30 | 2610723 | 1771311 | 2370684 |
| 0 | SRR1211274 | GATC | 293T | C | 30 | 2574521 | 1695417 | 2335329 |
| 0 | SRR1211275 | ACGT | 293T | A | 30 | 2301608 | 492050 | 1879325 |
| 0 | SRR1211275 | CTAG | 293T | B | 30 | 2616587 | 458868 | 2137945 |
| 0 | SRR1211276 | ACGT | 293T | A | 60 | 4503966 | 3525840 | 4322577 |
| 0 | SRR1211276 | CTAG | 293T | A | 90 | 3543837 | 2909141 | 3422767 |
| 0 | SRR1211276 | GATC | 293T | B | 60 | 3297393 | 2592298 | 3166286 |
| 0 | SRR1211276 | TGCA | 293T | B | 90 | 2576207 | 2070437 | 2482060 |
| 0 | SRR1211278 | GATC | HeLa | A | 90 | 2755908 | 2195256 | 2663203 |
| 0 | SRR1211278 | TGCA | HeLa | B | 90 | 3291760 | 2715574 | 3198220 |
| 0 | SRR1211278 | ACGT | 293T | C | 60 | 2867657 | 2301472 | 2785859 |
| 0 | SRR1211278 | CTAG | 293T | C | 90 | 3437232 | 2864421 | 3343478 |
| 0 | SRR1211279 | ACGT | HeLa | C | 90 | 1928866 | 1459999 | 1866066 |

In certain cases, setting the read quality to 63, as done by Porter et al., results in a very strong reduction in the number of reads. Therefore, we reduced the minimum read quality to 56.

## Reference library¶

The reference library contains all barcodes that, in the plasmid library sample, have a frequency of 0.000002 or larger and that are present in at least 2 replicates.

In [10]:

```
def make_reflib(qual,cell_line):
    minfreq = 0.000002
    samples = []
    i = 0
    for idx,row in metadata[metadata['cell line']==cell_line].iterrows():
        fn = '{}/noclustering/q={}/{}_{}.txt.gz'.format(datapath,qual,row['run accession'],row['tag'])
        data = {row[0]:int(row[1]) for row in csv.reader(gzip.open(fn),delimiter='\t') if not row[0].startswith('#')}
        nbc = np.sum(data.values())
        data_filtered = {key : val for key,val in data.iteritems() if val >= nbc*minfreq}
        samples.append(data_filtered)

    nbc = [len(s) for s in samples]
    reflib = []
    for bc in set([bc for s in samples for bc in s]):
        if np.sum([bc in s.keys() for s in samples]) > 1:
            reflib.append(bc)
    f = open('{}/noclustering/q={}/reflib_{}.txt'.format(datapath,qual,cell_line),'w')
    for bc in reflib:
        f.write(bc+'\n')
    f.close()
    
make_reflib(56,'plasmidlibrary')
```

## Analyze results with lentiviralbarcodes¶

We use the following procedure for all cell lines:

1. Search for runs and tags that contain data for given cell line
2. Read files with data for each replicate and population doubling:
   - Read barcodes and counts extracted from FASTQ files and only store barcodes that are present in the reference library.
   - When multiple files contain data for a single replicate and population doubling, the counts for identical barcodes are added.
3. Store data in an MxN array, with M the number of barcodes in the reference library and N the number of time points (4).

In [11]:

```
def read_counts(df,qual=56):
    f = open('{}/noclustering/q={}/reflib_plasmidlibrary.txt'.format(datapath,qual))    
    reflib = [line.strip() for line in f.readlines() if len(line) > 0]
    rawdata = np.zeros(len(reflib))
    for idx,row in df.iterrows():
        fn = '{}/noclustering/q={}/{}_{}.txt.gz'.format(datapath,qual,row['run accession'],row['tag'])
        if not os.path.isfile(fn):
            print 'could not find {}'.format(fn)
            continue
        reader = csv.reader(gzip.open(fn),delimiter='\t')
        csvdata = {row[0]:int(row[1]) for row in reader if (not row[0].startswith('#'))}
        for i,key in enumerate(reflib):
            if key in csvdata:
                rawdata[i] = rawdata[i]+csvdata[key]
    return rawdata

def read_cell_line(cell_line,qual=56,PDlist=None):
    f = open('{}/noclustering/q={}/reflib_plasmidlibrary.txt'.format(datapath,qual))    
    reflib = [line.strip() for line in f.readlines() if len(line) > 0]        
    md = metadata[metadata['cell line']==cell_line]
    cnts0 = read_counts(md[md['population doubling']=='0'],qual)
    if PDlist is None:
        PDlist = ['30','60','90']
    replicates = ['A','B','C']
    data = {}
    for rep in replicates:
        fn = '{}/{}_{}_noclustering_q={}.txt.gz'.format(datapath,cell_line,rep,qual)
        if os.path.isfile(fn):
            data[rep] = CloneData(np.loadtxt(fn),barcodes=np.array(reflib))
        else:
            df_rep = md[md['biological replicate']==rep]
            pddata = np.array([read_counts(df_rep[df_rep['population doubling']==PD],qual) 
                               for PD in PDlist])
            data[rep] = CloneData(np.concatenate(([cnts0],pddata)),barcodes=np.array(reflib))
            data[rep].save(fn)
    return data

def plot_exp_results(data,title=None):
    all_data = {rep : copy.deepcopy(d) for rep,d in data.iteritems()}
    all_data['mean']= data.values()
    fig = figure(figsize(4*w_im,.9*h_im))
    if title is not None:
        fig.suptitle(title)
    gs = gridspec.GridSpec(1,5)
    axes = [fig.add_subplot(ss) for ss in gs]
    
    plot_metric_as_bar(all_data, 'fleft', idx=[0,1,2,3], passages=[0,10,20,30],ax=axes[0],
                       show_legend=False, cmname='Set1', metric_name='% clones left',
                       keysorder=['mean','A','B','C'])    
    
    plot_fraction_clonesVSpop(data.values(),[0,1,2,3],['PD0','PD10','PD20','PD30'],
                          ax=axes[1],plot_sd=True,merge=False)
    axes[1].legend()
        
    plot_metric_as_bar(all_data, 'gini', idx=[0,1,2,3], passages=[0,10,20,30],ax=axes[2],
                       show_legend=False, cmname='Set1', metric_name='Gini coefficient',
                       keysorder=['mean','A','B','C'])        

    plot_metric_as_bar(all_data, 'nmajor', idx=[1,2,3], passages=[10,20,30],ax=axes[3],
                       show_legend=False, cmname='Set1', metric_name='major clones',
                   keysorder=['mean','A','B','C'])    
    
    plot_compare_replicates_single(data,abun=0.01,idx=[1,2,3],xtics=[10,20,30],
                                   fs=12,ax=axes[4],perc=True,showlegend=True);
    axes[4].set_xlabel('passage');
    axes[4].set_yticks(range(0,101,20));
    
    gs.tight_layout(fig, rect=[0, 0.03, 1, 0.95]) 

def save_pop_stats(data,fn):
    df = pd.DataFrame(columns=['passage','replicate','f25','f50','fleft','gini','nmajor'])
    passages = [0,10,20,30]
    for rep,cdata in data.items():
        df = df.append(pd.DataFrame(np.column_stack([passages,4*[rep],cdata.f25,cdata.f50,
                                cdata.fleft,cdata.gini,cdata.nmajor]),columns=df.columns))
    df.to_csv(fn,sep='\t',index=False,compression='gzip')
```

### K562¶

In [18]:

```
cell_line = 'K562'

data = read_cell_line(cell_line)
plot_exp_results(data,'K562 - no clustering - min_qual = 56')
```

### clonal K562¶

In [19]:

```
cell_line = 'clonalK562'

data = read_cell_line(cell_line)
plot_exp_results(data,'clonal K562 - no clustering - min_qual = 56')
```

### HeLa¶

In [20]:

```
cell_line = 'HeLa'

data = read_cell_line(cell_line)
plot_exp_results(data,'HeLa - no clustering - min_qual = 56')
```
